# Genome-wide analyses of Epstein-Barr virus reveal conserved RNA structures and a novel stable intronic sequence RNA

**DOI:** 10.1186/1471-2164-14-543

**Published:** 2013-08-09

**Authors:** Walter N Moss, Joan A Steitz

**Affiliations:** 1 Department of Molecular Biophysics and Biochemistry, Howard Hughes Medical Institute, Yale University School of Medicine, New Haven, Connecticut 06536, USA

**Keywords:** Epstein-Barr virus (EBV), Herpesvirus, RNA, RNA structure, Non-coding RNA (ncRNA), RNA-Seq, Bioinformatics, W repeat, sisRNA, RNA editing

## Abstract

**Background:**

Epstein-Barr virus (EBV) is a human herpesvirus implicated in cancer and autoimmune disorders. Little is known concerning the roles of RNA structure in this important human pathogen. This study provides the first comprehensive genome-wide survey of RNA and RNA structure in EBV.

**Results:**

Novel EBV RNAs and RNA structures were identified by computational modeling and RNA-Seq analyses of EBV. Scans of the genomic sequences of four EBV strains (EBV-1, EBV-2, GD1, and GD2) and of the closely related Macacine herpesvirus 4 using the RNAz program discovered 265 regions with high probability of forming conserved RNA structures. Secondary structure models are proposed for these regions based on a combination of free energy minimization and comparative sequence analysis. The analysis of RNA-Seq data uncovered the first observation of a stable intronic sequence RNA (sisRNA) in EBV. The abundance of this sisRNA rivals that of the well-known and highly expressed EBV-encoded non-coding RNAs (EBERs).

**Conclusion:**

This work identifies regions of the EBV genome likely to generate functional RNAs and RNA structures, provides structural models for these regions, and discusses potential functions suggested by the modeled structures. Enhanced understanding of the EBV transcriptome will guide future experimental analyses of the discovered RNAs and RNA structures.

## Background

Epstein-Barr virus (EBV) is widely disseminated in the human population. Upwards of 95% of the adult human population is infected with EBV [1]. EBV is implicated in a number of different cancers, including Hodgkin’s disease [2], nasopharyngeal carcinoma [3], hepatocellular carcinomas [4], lymphoepithelioma-like carcinomas [5], some breast cancers [6], and in autoimmune disorders such as Sjögren’s syndrome [7], dermatomyositis [8], lupus [9], rheumatoid arthritis [10], and multiple sclerosis [11]. EBV was the first cancer-associated virus to be discovered when in 1964 [12] it was isolated from tumors occurring in African children (Burkitt’s lymphoma [13]). Despite intense investigation for more than 50 years, the precise roles played by the virus in these diseases remains to be elucidated.

The ~170000 bp genome of EBV is a linear double-stranded (ds)DNA that circularizes to form an episome in the host cell nucleus. Infection occurs by entry of the EBV virion into human host epithelial cells and initially proceeds via an aggressive lytic phase. The virus migrates to B cells where it causes persistent lifelong infections marked by extended periods of latency with interspersed lytic reactivation [14]. EBV latency proceeds via three distinct programs, each expressing a different set of coding and non-coding viral gene products. Viral latent gene products rewire B cells to evade the host immune system and propagate the virus [15]. In a manner not yet fully understood, this rewiring increases the tumorigenic potential of EBV-infected cells, including making infected cells resistant to apoptotic pathways that would otherwise kill cancerous cells [16].

The EBV transcriptome is complex, consisting of multiple pre-mRNA transcripts that undergo extensive alternative splicing events and can, at various states of infection, yield different sets of gene products [17]. Two well-studied EBV-encoded ncRNAs (EBER1 and EBER2) are known to be expressed throughout infection [18], as are subsets of as many as 50 different EBV-encoded microRNAs (miRNAs) [19,20]. Although the precise functions of the EBERs have remained obscure, they are the most highly expressed RNAs in EBV-infected cells (~10^7^ copies per cell [18]). Roles in cancer have been proposed [21] and the EBERs are capable of inducing tumors in immune-suppressed mice [22]. Both EBERs form ribonucleoprotein complexes [23-26], including host proteins, and adopt well-defined secondary structures [27]. Except for the EBERs, RNA structure is a relatively understudied aspect of EBV virology; an analysis of RNA secondary structure in EBV could enable significant advances in understanding this important human pathogen.

RNA secondary structure plays important roles in many viruses, mediating functions as diverse as translation initiation [28], catalysis [29], viral genome packaging [30], alternative splicing [31,32], interactions with the host innate immune system [33], RNA lifetime/stability [34-36], and regulation of gene expression [37]. The discovery of RNA structure is an active area of research [38-40] and is aided by the availability of sophisticated non-coding (nc)RNA-discovery algorithms [41-43]. A successful strategy for discovering conserved RNA structures is implemented in the program RNAz [44]. In this approach, homologous sequences are aligned and divided into fragments. For each fragment, calculated parameters that include measures of the thermodynamic stability (z-score [45]) and conservation of secondary structure are used to make predictions based on a support vector machine (SVM) trained on known ncRNAs, which generates a probability classifier (p-class, scale of 0.0 to 1.0) for the fragment containing conserved secondary structure. The RNAz approach has been used to discover RNA secondary structures in a number of viruses [38,46-48].

## Results and discussion

### Global findings

**Figure 1:**
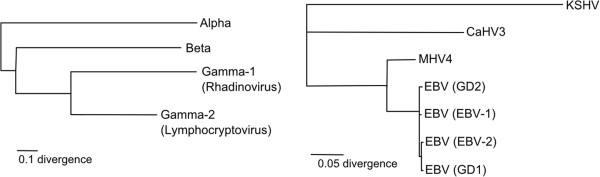
**Phylogenic relationships of analyzed viruses.** (Left) Phylogenetic tree showing the three herpesviridae sub-families (alpha, beta, and gamma), where the gamma sub-family is further divided into two genera: gamma-1 (Rhadinovirus) and gamma-2 (Lymphocryptovirus). The tree was generated from data in [51]. (Right) Breakdown of the gamma herpesvirus branch shown on the left. The consensus neighbor-joining tree (100 replicates) was based on the conserved herpesvirus DNA polymerase catalytic subunit (DPOL). Kaposi’s sarcoma-associated herpesvirus (KSHV), a gamma-1 herpesvirus, is used as an outgroup to illustrate the phylogenetic relationships between four EBV strains (EBV-1, EBV-2, GD1, and GD2) and two other lymphocryptoviruses used in this study [Macacine herpesvirus 4 (MHV4) and Callitrichine herpesvirus 3 (CaHV3)]. The tree was generated using the Geneious program.

RefSeq genomes (~170 kbp long) from four EBV strains (NC_007605.1, NC_009334.1, AY961628.3, HQ020558.1) and one from the closely-related (Figure 1) Macacine herpesvirus 4 (MHV4, NC_006146.1; also known as rhesus lymphocryptovirus) were aligned using the MAFFT alignment program [49,50]. The average pairwise sequence identity (APSI) of the alignment is 84.8%; the four EBV strains were close in sequence (APSI of 95.6%), while MHV4 had an APSI of 68.6% versus EBV.

Across the entire EBV genome the z-score average is -0.19 and the average p-class is 0.13. RNAz discovered 265 regions that map to known transcripts from the EBV genome (named locus 1 to 265 in Additional files 1 and 2) and have strong evidence of locally conserved and stable RNA structure. These regions (average length ~200 nt) are comprised of overlapping windows with high predicted probability of generating conserved RNA structure. The average p-class for the set of predictions was 0.91 with minimum and maximum values of 0.61 and 1.00, respectively (Additional file 1). The thermodynamic z-score is an important component in determining the p-class. This parameter reflects the thermodynamic stability of the native sequence predicted structure versus that of random sequence [45] (e.g. measures greater than expected folding stability). For predicted structured regions in EBV RNA, the average z-score was -2.38 with minimum and maximum values of -1.18 and -9.71, respectively. This analysis, however, does not account for long-range RNA interactions that may occur outside the overlapped prediction windows.

### Identification of known EBV ncRNAs

**Figure 2:**
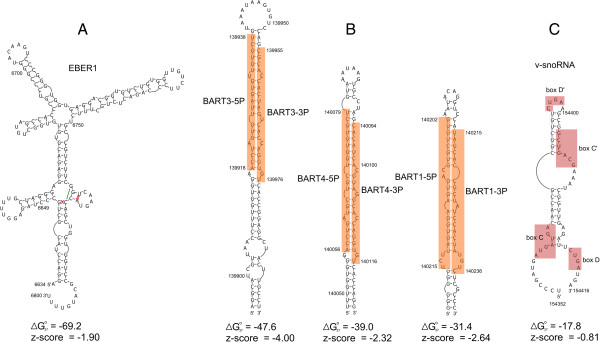
**Structure models for several known EBV ncRNAs.** Nucleotide positions for the EBV-2 RefSeq genome sequence are annotated on the structure. Below are the predicted Gibbs folding free energies (in kcal/mol) and z-scores. **(A)** EBER1 modeled structure. Base pairs that differ from the Rfam model (Rfam ID# RF01789) are covered by red Xs, while the mis-predicted Rfam base pair is indicated with a green line. **(B)** Modeled structures in RNAz-predicted structured region, locus 209, which contains the BART3, BART4, and BART1 miRNAs (indicated in orange boxes). Sequences are from the EBV-2 RefSeq (NC_009334.1) genome. **(C)** Modeled structure for the viral snoRNA (v-snoRNA) previously reported [52].

The genomic region specifying EBER1 occurs within locus 12 (Additional file 1). The structure model derived for EBER1 (Figure 2A) is almost identical to the model in the Rfam database (ID# RF01789). A single base pair in the Rfam model, between nt 6760 and 6770, is shifted to form a pair between 6646 and 6770, and the predicted closing AU pair of this hairpin is open in the Rfam model (Figure 2A). Additionally, C6649 is in a bulge-loop in the Rfam model, but the insertion of a G residue across from this nt allows a GC pair to form. EBER2, surprisingly, does not occur in an RNAz-predicted locus. The region containing EBER2 was not calculated to have a favorable z-score; however, this is most likely due to the inability of the primary sequence alignment to capture the conservation of structure for this RNA. EBER2 is less conserved in sequence than EBER1 (50.3% sequence conservation vs. 78.3% for EBER1) making primary sequence alignment difficult in this region. Common helices in EBER2 were not properly aligned, thus reducing the predicted free energy of the consensus structure, which resulted in an unfavorable z-score.

All but two of the EBV miRNAs annotated in the miRBase database [53] occur in RNAz-predicted structured regions. Each of the mature miRNA sequences, when mapped to predicted structure models, falls within a hairpin RNA structure that resembles a canonical pre-miRNA (Additional files 1 and 3). For example, Figure 2B shows three hairpins in locus 209, which include the BART3, BART4, and BART1 miRNAs. Each of these hairpins places the 5P and 3P mature sequences in canonical structural contexts: the mature miRNAs are offset and imperfectly base pair with each other. This is also true for the other 36 mature miRNA sequences that fall within predicted structured regions (Additional file 3).

Finally, a reported EBV-encoded viral small nucleolar RNA (v-snoRNA; [52]) also falls within an RNAz-predicted structured region (locus 235 in Additional file 1). The v-snoRNA corresponds to nt 154352 to 154416 (Additional file 3) and, in isolation, has a predicted folding free energy and z-score of -17.8 kcal/mol and -0.81, respectively (Figure 2C).

In addition to the regions corresponding to EBER1, the v-snoRNA, and 14 loci specifying EBV pre-miRNAs, there are 249 additional EBV regions with predicted conserved RNA structure (Additional file 1).

### Structure within introns

Sixty predicted structured regions overlap introns. Introns are a fertile source of structured ncRNAs [54,55] that play roles in a number of important biological processes: snoRNAs [56], miRNAs [57], and piRNAs [58] are expressed from introns. RNA structure within introns can alter accessibility or distance between functional elements and thus regulate splicing [59].

**Figure 3:**
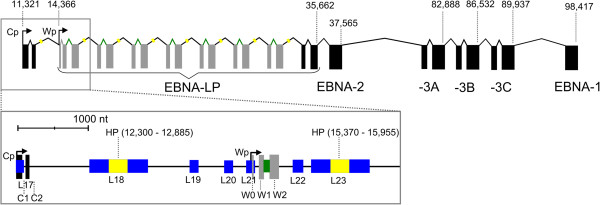
**Cartoon of the primary transcript encoding the EBNA proteins.** Exons are indicated with black or grey rectangles and the genomic location of the end of each protein-coding region is indicated above. The identical W1 and W2 coding exons that compose most of EBNA-LP are shown in grey. The Cp and upstream Wp promoter sites are indicated by bent arrows with the genome coordinates of the transcription start sites given above. The boxed region zooms in to show the 5′ end of the pre-mRNA, including the locations of the predicted long hairpin (HP) and ebv-sisRNA-1, colored yellow and green, respectively, with their genome coordinates given in parentheses. RNAz-predicted structured regions are shown in blue and labeled L17 to L23 for loci 17 to 23 (genome coordinates for these loci are in Additional file 1).

The most extensive regions of predicted structure in introns occur within a repetitive region of the EBV genome (the W repeats). The number of W repeats can vary, and the optimal number appears to be five to eight [60]. The W repeats are transcribed at the 5′ end of the long (~100000 nt) primary transcript encoding the Epstein-Barr nuclear antigen (EBNA) latent proteins (Figure 3). EBNA transcripts are particularly interesting because they have high nuclear-to-cytoplasmic abundance, suggesting non-coding nuclear functions [61].

Six EBNA proteins are expressed from mRNAs produced by extensive alternative splicing. Transcription of the EBNA RNA can occur from an upstream promoter, the C promoter (Cp), or a downstream W promoter (Wp) [62]. Early in latency III Wp is utilized, where splicing of W0 to W1 generates the start codon for EBNA-LP production [63]; later in latency III there is a switch to Cp where the non-coding C2 exon is joined to W1 to create the EBNA-LP start codon (Figure 3). Within the W repeats two short coding exons, W1 and W2, which are 66 and 132 nt long, respectively, are joined to form the EBNA-LP protein open reading frame (ORF) by removal of a long (2791 nt) and short (81 nt) intron. RNAz predicts widespread conserved and stable RNA structure covering 49% of the long W repeat intron. There are five predicted regions, which are identical in sequence in the W repeats (Figure 3): loci 18, 23, 28, 33, 38, 43, 48, and 53 are identical; loci 19, 24, 29, 34, 39, 44, 49, and 54 are identical; loci 20, 25, 30, 35, 40, 45, and 50 are identical; loci 21, 26, 31, 36, 41, 46, and 51 are identical; and finally, loci 22, 27, 32, 37, 42, 47, and 52 are identical. Loci 18, 19, and 20, although they are multiplied in the W repeats, are transcribed only when Cp is used to initiate transcription (Figure 3). Cp occurs just upstream of locus 17 and the C1 non-coding exon is within locus 17 (Figure 3).The first copy of Wp overlaps locus 21, which also contains W0 (Figure 3).

**Figure 4:**
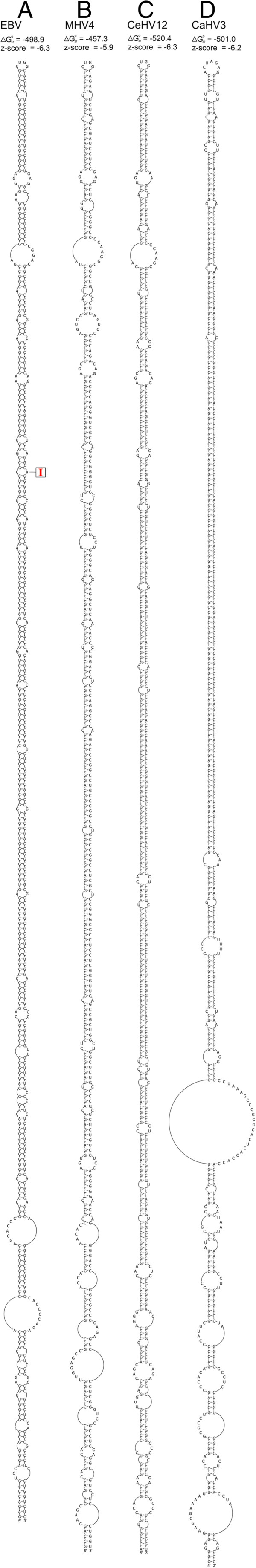
**Structure models for lymphocryptovirus repeat long hairpin RNAs.** A reported A-to-I editing site [66] is indicated with a red “I”. Shown are sequences from **(A)** Epstein-Barr virus (EBV), **(B)** Macacine herpesvirus 4 (MHV4), **(C)** Cercopithecine herpesvirus 12 (CeHV12), and **(D)** Callitrichine herpesvirus 3 (CaHV3).

Within loci 18, 23, 28, 33, 38, 43, 48, and 53 is a highly unusual modeled RNA structure, a very long (586 nt) tetraloop hairpin (Figure 4A). The INFERNAL package [64,65] was used to search for additional homologous structures. Three other lymphocryptovirus species [MHV4, Callitrichine herpesvirus 3 (CaHV3), and Cercopithecine herpesvirus 12 (CeHV12, also known as papiine herpesvirus)], members of the herpesvirus taxonomical group to which EBV belongs (Figure 1), possess homologous structures. These discovered hairpins occur within the long introns of repeats homologous to the W repeats of EBV, appearing six times in MHV4 (NC_006146.1 nt 12738 to 13335, 15820 to 16416, 18901 to 19497, 21982 to 22578, 25063 to 25659, and 28144 to 28740) and 4 times in CaHV3 (NC_004367.1 in the negative strand nt 120959 to 121293, 123871 to 124205, 126783 to 127117, and 129695 to 130029). Three hairpin homologs are found within the partial sequence of CeHV12 (AF200364.1 nt 6498 to 7112, 9542 to 10156, 12586 to 13200).

The APSI of these long hairpin sequences is 66.7%, with the CaHV3 sequence being most distant (APSI 45.1% versus EBV). The MHV4 and CeHV12 hairpins are most similar to EBV in sequence (APSI of 80.5% and 74.5%, respectively) and structure (Figure 4B and C and Additional file 4). The EBV, MHV4, and CeHV12 hairpins are each capped with a tetraloop closed by a GC pair (Figure 4A, B, and C) and only the first nt in the loop varies (UUGG, CUGG, and GUGG, respectively). The more distant CaHV3 hairpin, in contrast, is capped with a seven-nt loop (Figure 4D). Perhaps the identities of the capping nucleotides are of lesser importance to the function of this long hairpin than the overall conserved structure, which is marked by extensively base paired stem regions interspersed with internal (or bulged) loops (Figure 4). Internal loops may adopt interesting 2D and 3D structures; for example, the three-by-three internal loop that occurs 20 bps from the hairpin loop in EBV [Figure 4A, also conserved in MHV4 (Figure 4B)], may fold into a “3 RRs” motif [67] where the opposing internal loop nucleotides form consecutive sheared purine-purine pairs.

Additionally, global thermodynamic z-scores were calculated for each hairpin (Figure 4): all four have very negative z-scores (ranging from -6.3 to -5.9). Thus, the structures generated by these sequences are more than six standard deviations more thermodynamically stable than random sequences with the same dinucleotide content. This indicates that evolution is acting to preserve base pairing within each sequence, as well as the striking structural homology between these viral hairpins.

Very long RNA hairpins that are structurally similar to the EBV W repeat hairpin have been described in humans [68] and in *Caenorhabditis elegans*[69]. These structures are excellent substrates for adenosine deaminase acting on RNA (ADAR) editing enzymes, which convert adenosine to inosine (A-to-I editing). Interestingly, a previous study of A-to-I editing in EBV [66] found evidence for an editing site that maps to a region of the genome that we predict to be part of the long hairpin (Figure 4A). ADARs act on structured RNAs and have preferences for adenosines in particular sequence contexts [70], allowing the prediction of potential A-to-I editing sites and of the extent of editing. The reported editing site is predicted to be moderately edited (12.9% by ADAR2, Additional file 4) and is modeled to be in a CA internal loop (Figure 4A). There are additional sites predicted to be strongly edited in the EBV W repeat hairpin and in the homologous structures found in MHV4, CeHV12, and CaHV3 (Additional file 4). Few of these strongly predicted sites (seven), however, have apparent conservation (Additional file 4), which is unsurprising, as selection of ADAR sites may be partially stochastic [71].

RNA editing in introns, typically within Alu inserts [71], has been proposed to regulate RNA splicing [72]. Inosine can form wobble pairs with G and C residues and can contribute to RNA-protein interactions: thus A-to-I editing can alter important RNA-RNA and RNA-protein interactions [73]. Editing can alter splicing regulatory elements or create/destroy splice sites [72]. Perhaps editing of this long intronic hairpin also plays a role in splicing or in other functions post-splicing.

### Discovery of an EBV-encoded sisRNA

**Figure 5:**
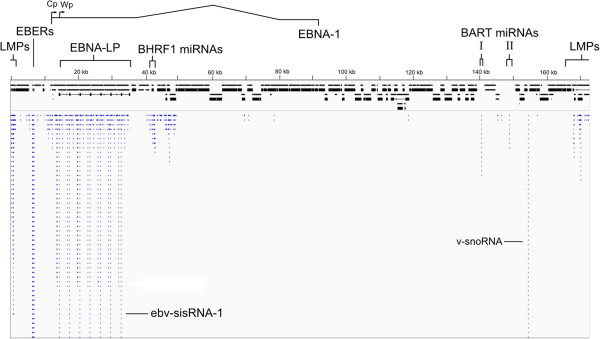
**Small RNA library RNA-Seq reads aligned with the EBV genome.** Reads are colored blue; the EBER1, EBER2, and sisRNA peaks are much larger than the represented area (see Table 1). A cartoon of the EBV genome is shown in black (exons as boxes and introns as thin lines). The locations of latently expressed genes [EBNA-LP, LMPs, EBERs (EBER1 and EBER2), BHRF1 miRNAs, and BART miRNAs (clusters I and II)] are indicated at the top of the figure, including the location of the Cp and first Wp promoter sites for producing mRNA for the EBNA-LP and five other EBNA proteins (additional details are in Figure 3). Peaks for the ebv-sisRNA-1 and v-snoRNA are indicated. Images were generated using the integrated genome viewer (IGV, [76,77]) with data from Additional file 5.

**Figure 6:**
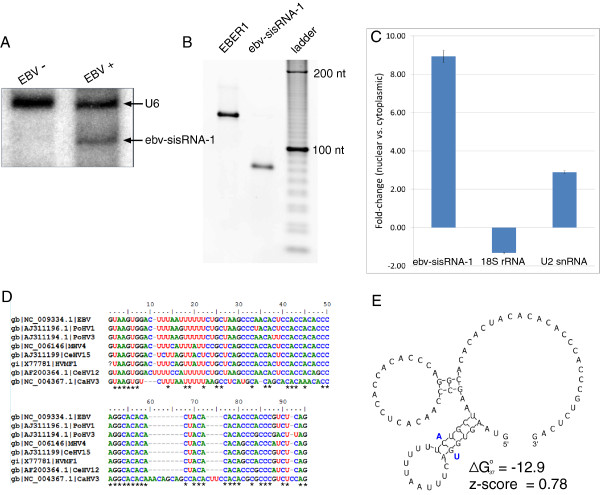
**Analyses of ebv-sisRNA-1. (A)** Northern blot for ebv-sisRNA-1 using RNA from EBV-negative BJAB cells and from isogenic EBV-positive BJAB-B1 cells. The bottom arrow points to the additional band observed in the EBV-positive BJAB-B1 lane that likely corresponds to the 81 nt ebv-sisRNA-1, while the upper arrow indicates the 106 nt human U6 snRNA (present in both cell types). **(B)** RT-PCR for EBER1 and ebv-sisRNA-1 using cDNA from BJAB-B1 nuclear RNA and primers complementary to the ends of EBER1 and the 5′ end plus nt 52 to 71 of ebv-sisRNA-1. **(C)** Fold-enrichment in nuclear versus cytoplasmic RNA as measured by RT-qPCR. Shown are the results for the sisRNA and control 18S and U2 snRNA, which are cytoplasmically and nuclearly enriched, respectively. Error bars indicate the standard error of three biological replicates, each with three technical replicates. **(D)** MAFFT sequence alignment of ebv-sisRNA-1 and those of seven other lymphocryptoviruses: Pongine herpesvirus 1 (PoHV1, AJ311196.1), Pongine herpesvirus 3 (PoHV3, AJ311194.1), Macacine herpesvirus 4 (MHV4, NC_006146), Cercopithicine herpesvirus 15 (CeHV15, AJ311199), Herpesvirus MF-1 from Macaca fascicularis (HVMF1, X77781), Cercopithecine herpesvirus 12 (CeHV12, AF200364.1), and Callitrichine herpesvirus 3 (CaHV3, NC_004367.1). The question mark at the first position in HVMF1 represents missing data, not a true gap. 100% conserved positions are indicated with stars below the alignment. **(E)** Predicted structure for ebv-sisRNA-1. Compensatory mutations in the upstream hairpin that convert a GC pair to an AU pair (in CaHV3) are indicated with bold blue nts. This sequence is repeated seven times in EBV: genome (NC_009334.1) coordinates 14603 to 14683, 17673 to 17753, 20743 to 20823, 23813 to 23893, 26883 to 26963, 29953 to 30033, and 33023 to 33103.

A small RNA library (30-200 nt size range) was constructed using nuclear RNA isolated from cultured human B lymphocytes stably infected with EBV (BJAB-B1 cells [74]). These cells exhibit a latency III program of gene expression, the most transcriptionally active program, which also produces latency I and II transcripts. Nuclear RNAs include not only EBER1 and EBER2 [75] but also intronic sequences that are excised during the splicing process. Unsurprisingly, the abundant EBER1 and EBER2 are present in the library and had the highest number of aligned RNA-Seq reads (Figure 5 and Table 1). A significant peak (~7.8% EBER1 abundance) corresponding to the v-snoRNA (Figure 5 and Table 1) was also observed. There was a surprisingly large peak originating from the W repeat region (Figure 5). This peak corresponds to reads that cover the entirety of the small intron (81 nt) that separates the W1 and W2 ORFs (Figure 3). Next to the EBERs, this was the largest peak observed. Comparing length-normalized reads between the intron and EBER1, the intron is estimated to be present at 21% the level of EBER1 or roughly on a par with EBER2, which is estimated to be 25% as abundant as EBER1 (Table 1). The presence of the intron in total nuclear RNA samples was confirmed by Northern blot (Figure 6A) and RT-PCR (Figure 6B). RT-qPCR then revealed that the intron is ~9-fold enriched in nuclear versus cytoplasmic RNA (Figure 6C).

**Table 1:** Regions with significant peaks in aligned RNA-Seq reads for undigested samples

	**5′ nt**	**3′ nt**	**Reads**	**Length (nt)**	% EBER1*
EBER1	6634	6800	1610	167	100.0
EBER2	6961	7133	415	173	24.9
ebv-sisRNA-1	-	-	162	81	20.7
v-snoRNA	154352	154416	49	65	7.8
Short intron	952	1025	41	74	5.7
Short intron	789	870	32	82	4.0
Short intron	459	539	21	81	2.7
Short intron	170112	170189	15	78	2.0

*Percent abundance vs. EBER1.

Stable intronic sequence (sis)RNAs have been described in Xenopus oocytes [78] and, interestingly, in herpes simplex virus-1 (HSV-1, an α-herpesvirus) [79] and in cytomegalovirus (a β- herpesvirus) [80]. In these other herpesviruses a stable intron, known as the latency-associated transcript (LAT) in HSV-1, is implicated in maintenance of latency [81]. The EBV sisRNA (ebv-sisRNA-1) is the first of this class of ncRNAs to be described in EBV. The ebv-sisRNA-1 differs from the LAT in two major respects: the LAT is much larger (>2 kb), and the functional form of the LAT is believed to be the lariat-intron splicing intermediate, whereas the ebv-sisRNA-1 is likely to be a linear molecule. For both ends of ebv-sisRNA-1 to have been free for ligation in the small RNA library construction step, the 5′ end of the released intron could not have been sequestered in a lariat structure. This suggests that ebv-sisRNA-1 has been acted upon by the debranching enzyme, which also linearizes intron-derived snoRNAs during the maturation of this important class of ncRNAs [82].

Introns homologous to ebv-sisRNA-1 were found in other lymphocryptoviruses (Figure 6D). The ebv-sisRNA-1 sequence and these RNAs have an APSI of 83.3%, with the CaHV3 sequence being most distant (57.6% APSI). In all analyzed EBV strains (EBV-1, EBV-2, GD1, and GD2), the 81-nt ebv-sisRNA-1 sequence is 100% conserved, whereas the APSI of the genome sequences of these strains is 95.6%. Within the CA-rich region an 11-nt sequence, from positions 49 to 59, is 100% conserved in the analyzed lymphocryptoviruses (Figure 6D). The distribution of dinucleotide frequencies in the ebv-sisRNA-1 sequence is skewed, with CA being the most frequent, followed by AC and CC. The presence of CA-rich regions in the sisRNA is interesting as such sequences are able to bind hnRNP L protein and modulate splicing [83].

Although ebv-sisRNA-1 did not overlap an RNAz-predicted region, the secondary structure for this RNA was modeled on the basis of the eight sequences shown in Figure 6E. Two small hairpins are predicted (Figure 6E): an upstream hairpin that places the U-rich motif in a loop and a weak (3 base pair) hairpin in the CA-rich region. A compensatory mutation (double point-mutation that preserves base pairing) is observed in the more stable upstream hairpin (Figure 6E, a conserved GC pair is converted to a UA pair in CaHV3), which suggests that evolution is acting to preserve this structure. It is interesting to find evidence of a conserved hairpin that places the U-rich motif in a loop, as U-rich sequences often bind proteins [84] and hairpins can pre-organize RNA sequence motifs for RNA-protein interactions [85]. The CA-rich region is likely unstructured and free to associate with other interactors.

### Additional putative sisRNAs in EBV

Other than the W repeat sisRNAs, EBV possesses 18 additional short introns (Table 2) interspersed throughout its genome. These short introns range in conservation from 82.9% to 100.0% APSI (average of 97.2%) and are predicted to fold into structures with a range of thermodynamic stabilities (from -0.4 to -59.6 kcal/mol). Four sequences have significantly negative z-scores [< -1.0, from nt 11466 to 11610, 80362 to 80446, 87014 to 87087, and 105929 to 106056 (Table 2)] and one intron has a moderately favorable z-score (-0.54) from nt 1496 to 1573. These short introns also overlap with RNAz-predicted structured regions (Table 2).

**Table 2:** EBV short intron predicted free energies and z-scores

**5′ nt**	**3′ nt**	**Length**	**APSI**^**a **^**(%)**	**ΔG (kcal/mol)**	**Z-score**	**RNAz**
273	359	87	99.4	−11.9	0.77	
459	539	81	96.9	−15.1	0.56	
789	870	82	100.0	−12.7	0.25	
952	1025	74	100.0	−3.0	1.12	
1197	1279	83	100.0	−10.9	0.24	
1496	1573	78	99.4	−20.7	−0.54	Locus 2
11466	11610	145	99.2	−41.3	−3.20	Locus 17
14939	14541	149	98.2	−30.7	1.45	
14603^b^	14683^b^	81	100.0	−12.9	0.78	
35474	35557	84	97.0	−4.8	0.8	
71968	72073	106	100.0	−42.6	−0.41	
80362	80446	85	94.3	−23.1	−1.12	Locus 113
83431	83508	78	97.0	−9.3	1.13	
87014	87087	74	99.3	−25.6	−1.88	Locus 126
90856	90940	85	95.9	−14.5	−0.02	
91046	91196	151	82.9	−47.3	1.06	
105929	106056	128	96.2	−59.6	−3.52	Locus 152
169949	170024	76	95.0	−3.3	0.64	
170112	170189	78	98.3	−0.4	0.22	

^a^APSIs calculated for four EBV strains (EBV-1, EBV-2, GD1, and GD2).

^b^Nucleotide numbers for first instance of ebv-sisRNA-1.

**Figure 7:**
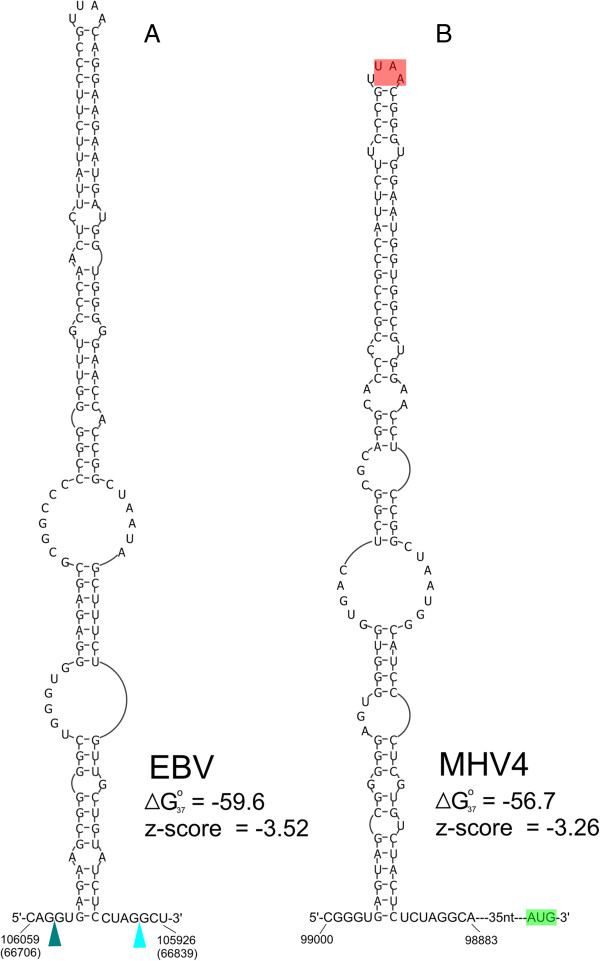
**Hairpin structure predicted in BBLF2/3 mRNA. (A)** Hairpin modeled for the EBV BBLF2/3 short intron. Splice donor and acceptor sites are indicated with dark and light blue arrows, respectively. Nucleotide numbers in parentheses correspond to the reverse genome strand models in Additional file 3 (locus 152). The predicted free energy (in kcal/mol) and z-score of the hairpin are shown. **(B)** Homologous hairpin structure in MHV4. The BBLF2 translation stop codon is shaded in red and the BBLF3 start codon is shown in green.

Particularly interesting is the sequence from nt 105929 to 106056, which has the most favorable predicted folding free energy and z-score of any EBV intron (Table 2). This intron occurs within the lytically-expressed BBLF2/3 gene, which generates part of a helix-primase complex essential for replication of the EBV genome [86]. The modeled secondary structure of the intron is shown in Figure 7A; it forms a tetraloop hairpin that includes all of the intron sequence. This intron structure is contained within RNAz-predicted locus 152, where the basal stem of the hairpin extends into coding regions by 25 nt (Additional file 3, nt 66692 to 66844 in the reverse genome sequence) and the splice donor and acceptor are base paired within helices. A similar hairpin structure, identified by INFERNAL, occurs in the MHV4 genome (Figure 7B). Rather than being joined together via splicing, MHV4 BBLF2/3 is translated from two ORFs that produce BBLF2 and BBLF3 as separate proteins. This same intronic region in EBV partially codes for BBLF2 in MHV4 (the translation stop codon occurs within the UUAA tetraloop) and is also present in the 5′ UTR of BBLF3 (Figure 7B). It is remarkable that the hairpin structure has been maintained evolutionarily between EBV and MHV4 despite different apparent roles in protein expression: in EBV it brings splice sites closer together in space and may be processed out to form an independent structured RNA, whereas in MHV4 it presents a stop codon in a loop and is not processed out of the mRNA.

Four EBV short introns have significant numbers of mapped reads in the small RNA library (Table 1); however, they are lower in abundance than ebv-sisRNA-1. In the EBV latent membrane protein (LMP-2B) gene, for example, there are three short introns with significant numbers of aligned read peaks (nt 459 to 539, nt 789 to 870, and nt 952 to 1025 in Table 1). Indeed, the intron encompassing nt 952 to 1025 has an abundance of ~5.7% relative to EBER1, which is similar to the value observed for the v-snoRNA (7.8%, Table 2). The short introns in EBV, including those that are expressed during latency III and those with few or no mapped reads, may represent a family of sisRNAs. It will be interesting to learn if they are differentially expressed in other viral latency programs or during the lytic phase. Short introns are an attractive source of small ncRNAs, as they are released as by-products of pre-mRNA splicing.

### Structure overlapping splice sites

The importance of RNA structure in the regulation of splicing has been highlighted in two excellent reviews [59,87]. Structure can be inhibitory, by sequestering splice sites [88] or regulatory elements [89], or it can enhance splicing by presenting sites in an accessible conformation [90] or by bringing splice sites into closer proximity with each other [91]. Conformational switching between accessible and inaccessible structures may also regulate splicing [92].

**Figure 8:**
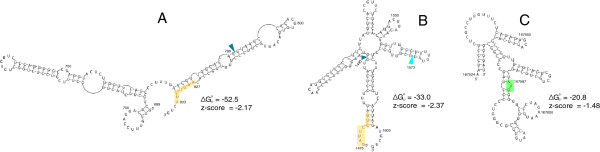
**Predicted RNA structures containing splice sites and a start codon. (A)** Structure model for RNAz-predicted structural region locus 1 (in LMP-2B gene). The splice donor site is indicated with a dark blue colored wedge and a putative intronic splicing enhancer is colored yellow. **(B)** Structure model for locus 2 (in the LMP-2B gene). The splicing acceptor site is indicated with the light blue wedge and the yellow box now indicates a putative exonic splicing enhancer. **(C)** Structure model for locus 263. The start codon for latent membrane protein 2A (LMP-2A) is indicated by the green box.

In EBV, there are seven predicted structured regions that include splice sites (Additional file 1). Of these, three include a splice donor or acceptor, while the remaining regions include both sites. For example, in the LMP-2B gene, locus 1 overlaps a splice donor site (after nt 788, Additional file 3), which is buried within the stem of a tetraloop hairpin; towards the 3′ end of this structure (nt 827 to 833) is a putative intronic splicing enhancer (Figure 8A). Locus 2 spans an entire intron and includes both the donor and acceptor sites (after nt 1495 and 1573, respectively, in Additional file 3). Additionally, there is a putative exonic splicing enhancer at the beginning of locus 2 (nt 1475 to 1482). The modeled structure for locus 2 (Figure 8B) organizes this region into a complex multibranched structure where the splice donor and acceptor sites are each in helices: the donor at the end of the basal stem and the acceptor within a hairpin loop. Interestingly, each splice site occurs directly after or near a predicted UU/UU two-by-two internal loop motif, in which UU mismatches maintain stable [93,94] helical 3D conformations [95]. Internal loops and non-canonical pairs can present complex patterns of chemical functional groups in protein-accessible regions of a folded RNA [96], which may influence the binding of splicing factors. Additionally, the 3D folding of this structure may bring splice sites close together .

### Structure overlapping coding regions

Many (150) predictions overlap EBV coding regions, in some cases including a significant portion of an ORF. In the lytically-expressed EBV FGAM-synthase gene, for example, predictions cover 40% the coding nt (locus 3 to locus 9; Additional file 1). More dramatically, the BGLF3 gene overlaps four predicted structured regions (locus 163 to locus 166; Additional file 1), which span 84% of the coding region including part of the start codon. Interestingly, many viral genomes appear to be enriched for RNA secondary structure [97,98], including within coding regions [38,99,100]. RNA structure in coding regions can have a variety of functions [101], such as altering reading frames via frame-shifting pseudoknots [37] or by inducing ribosomal pausing [102], which can impact numerous co-translational processes [103,104]. The identification of conserved and stable RNA secondary structures within EBV coding regions suggests that they may play important roles in this virus.

Twenty-one predicted structured regions include translation start or stop codons. Locus 263, for example, includes the start codon for the latent membrane protein 2A (LMP-2A), which is involved in the regulation of host B-cell signaling and is implicated in EBV-associated tumorigenesis [105]. The first 63 nt of locus 263 fall within the 5′ UTR of the LMP-2A mRNA (Figure 8C). The start codon is predicted to occur within the stem helix of a multibranch loop. RNA structure can play a role in regulating translation through modulating the accessibility of codons. Strong RNA structure at stop codons, for example, can stimulate stop codon read-through [106], whereas modulation of start codon accessibility can affect translational efficiency [107]. Perhaps the stable structure (z-score -1.48, Figure 8C) that encompasses the LMP-2A start codon modulates the expression of this important gene.

## Conclusion

Bioinformatics analyses of the EBV genome revealed 265 regions with putative conserved RNA structure. The computational screen successfully identified most of the known EBV ncRNAs: EBER1, a virally-encoded snoRNA, and 42 miRNAs. Other predicted structured regions of the EBV genome occur at or near sites that suggest potential functions for RNA structures. A very long hairpin structure in the W repeat region that is conserved in lymphocryptoviruses contains at least one A-to-I editing site, with possible implications for splicing. Additional regions span splice sites or start and stop codons. These structure predictions may prove useful since RNA and RNA structures are attractive targets for chemotherapy [108-111]. The modeled RNA structures are rich in internal loops, e.g. the UU/UU internal loops near two splice sites in LMP-2B, which are potentially drugable structural motifs [112]. RNA splicing, in particular, is an attractive target for RNA therapeutics [113,114].

RNA-Seq data identified several putative sisRNAs, including one in the W repeat region (ebv-sisRNA-1) that is abundant and localizes in the nucleus. These results lay the groundwork for future experiments to further characterize the proposed structures and to understand the functional significance of the discovered RNAs and RNA structures in EBV. All structural data are available in the Additional files. In addition, data for the W repeat long hairpin and ebv-sisRNA-1 will be deposited in the Rfam database.

## Methods

### Structure prediction and modeling

EBV RefSeq genomes from four different viral strains, EBV-1 (NC_007605.1), EBV-2 (NC_009334.1), GD1 (AY961628.3), and GD2 (HQ020558.1), plus one closely-related genome from Macacine herpesvirus 4 (NC_006146.1), were obtained from the NCBI nt database. Genome sequences were aligned using the MAFFT program [49,50] implementing the FFT-NS-1 alignment strategy. The alignment was processed into 120-nt windows with a 10-nt step-size using the Perl script rnazWindow.pl provided with the RNAz program. Predictions of windows with likely conserved and stable RNA structure were made for both the plus and minus strands with the RNAz 2.1 [42,44] program using a dinucleotide randomization model. The RNAz output was processed to combine windows with higher probability of structure (p-class > 0.5) into loci. These loci were further filtered to include only those that had at least one window with p-class > 0.9. When predictions had ambiguous strand orientations, e.g. occurred in a genome location that could be transcribed from either strand, the program RNAstrand [115] was used to determine if structure was more likely to be in the forward or reverse strand.

From each predicted locus the EBV-2 sequence was extracted and used in a BLAST [116] search against the NCBI nt database. The returned homologous sequences were compiled and each locus was aligned using the MAFFT program implementing the Q-INS-i method. Initial models for these alignments were made using RNAalifold [117]. Alignments and predicted consensus structures were converted to Stockholm format alignments and used to build covariance models with the INFERNAL package [64]. These covariance models were used to search for potentially homologous sequences/structures in a database of all available herpesvirus RefSeq genomes. Returned sequences were added to the alignments and structural predictions were made using RNAalifold as well as the program TurboFold [118] followed by manual model refinement. Resulting models were used to perform additional iterative INFERNAL searches and structure revisions until no further model improvement could be made (finding the structure that was best conserved in the alignment).

2D renderings of modeled RNAs were generated using the PseudoViewer program [119] and manually processed using the open-source graphics program Inkscape and ImageJ [120,121]. The Gibbs free energy of folding at 37°C [122,123] for models was calculated using the efn2 method implemented in the RNAstructure package [124]. The z-scores for sequences were calculated by generating sets of 200 dinucleotide randomizations using the SIMMONICS program [125] and then calculating energies with RNAfold [126]. The difference in native free energy versus the randomized set was normalized by the standard deviation to give the z-score.

### Small RNA-Seq analysis

EBV-positive BJAB-B1 cells were maintained at 37°C and 5% CO_2_ in RPMI media supplemented with FBS (10%) and penicillin [20]. Cells that had been expanded from 1 ml frozen stock to 200 ml culture volume were grown for an additional 48 hrs and were then pelleted by centrifugation at 1000 × g for 5 min at 4°C. Pellets were washed 3× with ice-cold PBS buffer (plus DTT). The pellet, on ice, was resuspended in cell disruption buffer [127] (10 mM KCl, 1.5 mM MgCl2, 20 mM Tris-Cl, 1 mM DTT, 0.1% Triton-X) and moved to a chilled Dounce homogenizer (Kontes, type B). Homogenization proceeded on ice until mostly free nuclei were observed under microscope. The lysate was transferred to centrifuge bottles and spun at 1500 × g for 5 min at 4°C. The supernatant, containing the cytoplasmic fraction, was removed to a fresh tube and a 3× volume of Trizol reagent was added. The nuclear fraction containing pellet was dissolved in 3 ml of Trizol reagent and both fractions were flash frozen in a dry-ice/ethanol bath and stored at -80°C overnight. Each sample was chloroform extracted and then precipitated at room temperature for 10 min using isopropanol. RNA was pelleted by centrifugation at 10000 × g for 10 min at 4°C. Pellets were resuspended in water and treated with RNase-free DNase I according to manufacturer’s protocol (Promega). RNA was further purified/recovered by RNeasy RNA purification kit (Qiagen). Samples were treated again using DNase I to remove any traces of genomic DNA contamination and recovered using the RNeasy kit. RNA integrity was checked using an Agilent BioAnalyzer: the RNA integrity number for the undigested samples was equal to 10, indicating that sample annealing did not degrade the RNA. RNA samples were used to build libraries using a small RNA library kit (Illumina), where specific adapters were ligated to the 5′ and 3′ ends, before barcoding and cDNA synthesis. The cDNA was fragmented and size-selected to contain fragments in the range of 30 to 200 bp. RNA-Seq data, 75 bp reads, were acquired on an Illumina HiSeq instrument run in paired-end mode. Reads were aligned to the EBV-2 RefSeq genome (NC_009334.1) using the Bowtie 2 program [128] (data given in Additional file 5).

### Analysis of ebv-sisRNA-1 expression

20 μg of total RNA from EBV-positive BJAB-B1 and EBV-negative (but otherwise isogenic) BJAB cells were fractionated by denaturing 10% PAGE. RNA was transferred to an Amersham Hybond-N + positively charged nylon membrane then cross-linked to the membrane using UV radiation. The membrane was incubated at 37°C for 1 hr in pre-hybridization buffer (GE) and washed 3× in double-distilled water (ddH_2_O). The membrane was then incubated overnight at 37°C with 5′-radiolabeled DNA probe complementary to nts 37 to 66 of the 106 nt human U6 snRNA (NR_046494.1), probe sequence: 5′-GCAGGGGCCATGCTAATCTTCTCTGTATCG-3′. The blot was washed 3× in wash buffer (0.005% SDS and 3× SSC), wrapped in plastic film, exposed to a phosphor screen for 16 hrs and imaged on a Storm phosphorimager. The blot was rehybridized with 5′-radiolabeled DNA probe complementary to nt 19 to 46 of ebv-sisRNA-1, probe sequence: 5′- TGTGGTGGAGTGTTGGGCTTAGCAGAAA -3′.

For the RT-PCR and RT-qPCR analyses, 2 μg nuclear or cytoplasmic RNA were used to generate cDNA with the High Capacity cDNA synthesis kit (Applied Biosystems). 5 μl 100× diluted cDNA was used for PCR with EBER1 primers complementary to the transcript’s 5′ and 3′ ends: FWD 5′-AGGACCTACGCTGCCCTAGA-3′ REV 5′-AAAACATGCGGACCACCAGCTGG-3′, and ebv-sisRNA-1 primers complementary to the 5′ end and nt 52 to 71: FWD 5′-GTAAGTGGACTTTAATTTTTTCTGCTAAGCCC-3′ REV 5′-TGGGTGTGTGTAGTGTGTGC-3′. Template was combined with 5 μl 10× PCR buffer (NEB), 1 μl 10 mM dNTPs, 1 μl each of 10 μM primer and 0.25 μl Taq (NEB) and ddH_2_O to a final volume of 50 μl. The thermocycler protocol was: (i) initial denaturation at 90°C for 10 min, (ii) melt at 90°C for 15 s, (iii) anneal at 53°C for 30 s, (iv) extend at 60°C for 30 s, (v) cycle back to step ii 40×. Amplification products were analyzed by denaturing 10% PAGE and stained with ethidium bromide. Amplicon sizes were determined by comparison to a 10 bp ladder (Invitrogen).

For RT-qPCR a master mix was made of FastStart Universal SYBR Green Master Mix (Roche), ddH_2_O and each primer (final concentration of 300 nM). 45 μl aliquots were added to a 96-well plate before adding 5 μl 100× dilute cDNA (three biological replicates, each run with three technical replicates, including no template and reverse transcriptase controls). In addition to the ebv-sisRNA-1 primers, amplification of nt 8 to 104 of the nuclear-enriched U2 snRNA (NR_002716.3) used primers: FWD 5′-CTCGGCCTTTTGGCTAAGAT-3′ REV 5′-TATTCCATCTCCCTGCTCCA-3′; and nt 994 to 1071 of the cytoplasmically-enriched 18S rRNA (NR_003286.2) used primers: FWD 5′-CGAAAGCATTTGCCAAGAAT-3′ REV 5′-GCATCGTTTATGGTCGGAAC-3′. The same thermocycler protocol used for PCR was used for the qPCR run, and data was collected on an Applied Biosystems StepOnePlus Real-Time PCR system. Data were exported to Microsoft Excel and analyzed using the Delta Ct method to find the ratio of nuclear to cytoplasmic abundance.

## Abbreviations

APSI: Average pairwise sequence identity; CaHV3: Callitrichine herpesvirus 3; CeHV12: Cercopithecine herpesvirus 12; EBV: Epstein-Barr virus; MHV4: Macacine herpesvirus 4; miRNA: MicroRNA; ncRNA: Non-coding RNA; nt: Nucleotide(s); sisRNA: Stable intronic sequence RNA; snoRNA: Small nucleolar RNA; v-snoRNA: Viral snoRNA; EBNA: Epstein-Barr nuclear antigen; Cp: C promoter; Wp: W promoter; ORF: Open reading frame; ADAR: Adenosine deaminase acting on RNA; LAT: Latency associated transcript; EBER: Epstein-Barr virus encoded RNA.

## Competing interests

The authors declare that they have no competing interests.

## Authors’ contributions

WNM: design and execution of experiments, data analysis/interpretation, and manuscript preparation. JAS: data analysis/interpretation, manuscript preparation, and final approval of the version to be published. All authors read and approved the final manuscript.

## Supplementary Material

Additional file 1 **Table showing RNAz-predicted regions with likely stable and conserved RNA structure.** Annotated regions (e.g. splice sites and miRNAs) are highlighted in different colors, which match color-annotated structure models provided in Additional file 3 with start and stop codons in green and red, respectively; splice sites in blue; miRNAs in orange; the W repeat hairpin in yellow; EBER1 in purple; and the v-snoRNA in brown. The columns for the 5′ and 3′ nt are the first and last nt of the EBV genome (NC_009334.1) that comprise the predicted structured region. The 5′ and 3′ nt-Rev numbers indicate the location of structure that likely occurs in RNA transcribed from the reverse genome sequence (corresponding to the structure models shown in the REV_STR worksheet in Additional file 3). Click here for file

Additional file 2 BED file with locations of RNAz-predicted structured regions. Click here for file

Additional file 3 **Spreadsheet showing structure models for predicted EBV structured regions.** The sequence shown is for the EBV-2 strain (NC_009334.1). In EBV, transcription can occur from either DNA strand and results for the forward and reverse sense genome sequences are presented in separate worksheets (FWD_STR and REV_STR). RNA structures are in “dot-bracket” notation, with paired sites indicated with matched brackets and unpaired with dots. Colored regions correspond to annotated RNAs in Additional file 1. Click here for file

Additional file 4 **Structure models and predictions of possible A-to-I editing sites (using the Inosine Predict program **[70]**) for lymphocryptovirus repeat long hairpin RNAs.** Each virus has a separate listing with nt position numbered for just the hairpin, followed by the sequence, the secondary structure in dot-bracket notation, the calculated percent editing for four ADAR specificities, and the maximum predicted value of all ADAR specificities. A reported A-to-I editing site [66] is colored red. Click here for file

Additional file 5 BED file with aligned RNA-Seq reads from BJAB-B1 nuclear small RNA sample. Click here for file
